# Clinicopathological and prognostic value of preoperative plasma fibrinogen in gastric cancer patients: A meta-analysis

**DOI:** 10.1097/MD.0000000000017310

**Published:** 2019-10-04

**Authors:** Fei Cheng, Chunyan Zeng, Ling Zeng, Youxiang Chen

**Affiliations:** Departments of Gastroenterology, The First Affiliated Hospital of Nanchang University, Nanchang, Jiangxi, China.

**Keywords:** gastric cancer, meta-analysis, plasma fibrinogen, prognosis

## Abstract

**Background:**

Increasing evidence has revealed that plasma fibrinogen may serve as a prognostic indicator in multiple malignancies. However, there have been some conflicting findings on the prognostic value of plasma fibrinogen in gastric cancer (GC). We conducted a meta-analysis to explore the correlation between plasma fibrinogen and clinic outcome in GC.

**Methods:**

A comprehensive literature search was conducted using the Embase, the Web of Science, the Cochrane library, and PubMed databases. Combined hazard ratios (HRs) and odds ratios (ORs) with 95% confidence intervals (CIs) were used to investigate the impact of elevated plasma fibrinogen on the prognosis and clinicopathological features of patients with GC.

**Results:**

A total of 11 studies involving 8315 patients were selected for this meta-analysis. The pooled results suggested that elevated plasma fibrinogen in GC patients was related to worse overall survival (OS) (HR = 1.57, 95% CI: 1.36–1.81, *P* < .001) and recurrence-free survival (RFS) (HR = 2.54; 95% CI: 1.19–5.41, P = .016). Additionally, a high level of fibrinogen was closely correlated with advanced tumor stage (OR = 2.14, 95% CI: 1.83–2.50, *P* < .001), lymph node metastasis (OR = 1.81, 95% CI: 1.56–2.11, P < .001), distant metastasis (OR = 1.48, 95% CI: 1.12–1.94, P = .005), deeper tumor invasion (OR = 2.25, 95% CI: 1.47–3.45, *P* < .001) and high carcinoembryonic antigen (OR = 1.41, 95% CI: 1.18–1.68, *P* < .001). However, there was no significant association between plasma fibrinogen and the differentiation grade (OR = 1.00, 95% CI: 0.86–1.17, *P* = .967). The Egger regression test indicated evidence of publication bias for OS.

**Conclusion:**

Elevated plasma fibrinogen could be a potential predictor for worse OS and RFS in GC patients and a significant risk factor associated with aggressive clinical features.

## Abstract 2

Supplemental Digital Content is available in the text

## 1 Introduction

Although the incidence of gastric cancer (GC) is declining, it remains the fifth most common malignancy, with a very high cancer-related global mortality.^[1]^ Radical gastrectomy without residual tumor is still the most effective treatment for GC patients. Nevertheless, many patients are diagnosed initially at advanced or metastatic disease stages, which greatly attenuates the survival benefits of surgery. Moreover, the outcome of GC patient who received radical surgery is not satisfactory, and at least 50% of these patients experience a lethal relapse within 5 years.^[2–4]^ At present, the tumor-node-metastasis (TNM) staging system has been one of the most useful indicators for predicting prognosis in GC. However, TNM staging is often performed after tumor resection and depends on the reliable pathological diagnosis. Hence, it is necessary to investigate easily accessible biomarkers for accurate prognosis prediction.

Inflammation and blood coagulation disorders are frequently observed in patients with malignant tumors.^[5–8]^ Fibrinogen, a vital clotting factor that is closely correlated with the degree of inflammatory response, has a substantial impact on carcinogenesis and tumor progression.^[9,10]^ Furthermore, an elevated plasma fibrinogen level has been reported in various types of cancer and is involved in advanced clinical stages, distant metastasis and postoperative recurrences. In recent years, a number of studies also demonstrated the association between plasma fibrinogen and clinic outcomes in GC, but the results remain inconclusive. For instance, Song et al^[11]^ verified that fibrinogen levels in GC are significantly higher than those in benign gastric tumors, and are implicated in an unfavorable prognosis. Other findings also revealed that plasma fibrinogen is an important prognostic factor for predicting poor overall survival in GC patients.^[12,13]^ However, studies by Wakatsuki and Zhang failed to confirm a definitive relationship between preoperative plasma fibrinogen and worse outcomes in GC.^[14,15]^

Therefore, we conducted a comprehensive and systematic meta-analysis to determine the prognostic value of plasma fibrinogen in patients with GC. In this study, we also investigated the correlation between high plasma fibrinogen and the clinicopathological features of GC.

## 2 Materials and methods

### 2.1 Literature search

This meta-analysis was conducted based on the Preferred Reporting Items for Systematic Reviews and Meta-Analyses Protocols (PRISMA) statement.^[16]^ For this study, we retrieved relevant studies that were published before February 24, 2019 from the Embase, the Web of Science, the Cochrane library and PubMed databases. The MeSH search terms and text words were as follows: “stomach neoplasm” or “gastric neoplasm” or “gastric cancer” or “gastric adenocarcinoma” or “gastric carcinoma” or “cancer of the stomach”, and “fibrinogen” or “plasma fibrinogen”, and “prognosis” or “prognostic” or “survival” or “outcome”. The references of eligible studies were also searched manually for all available studies. Two investigators (CYZ and LZ) independently conducted the literature search. Ethical approval and patient consent were not applicable as this study is a meta-analysis based on published studies.

### 2.2 Study selection criteria

The eligible studies met the following criteria: the fibrinogen level was detected in plasma samples before surgery; information on the hazard ratio (HR) with the 95% confidence interval (CI) were provided, or there was available data that allowed manual calculation of these values; the association between fibrinogen and the prognostic value or/and clinical features in GC patients was evaluated; the studies were published only in English. Studies were excluded if they met any of the following criteria: duplicate publications or overlapping studies; conference abstracts, reviews, case reports, meta-analysis; non-English articles; insufficient data to extract or calculate HRs with 95% CIs.

### 2.3 Data extraction and quality assessment

Two investigators (FC and CYZ) independently reviewed all of the included studies and extracted the available data. Any disagreement was resolved by consensus involving a third investigator (**YXC**). The extracted information in each study included: the first author's name, publication year, study design, study country, age of patients, gender of patients, sample size, tumor stage, metastasis status, cut-off value, outcome measures, follow-up time, detection method, HR with 95% CI and relevant clinicopathological data. If data for HRs with their 95% CI was not directly obtained, we extracted these values from Kaplan–Meier survival curves using Engauge Digitizer software version 4.1.^[17]^ If both univariate and multivariate analyses were reported in a study, the multivariate result was selected. Furthermore, the Newcastle–Ottawa Scale (NOS) was used for quality assessment of the included studies, with scores ranging from 0 to 9.^[18]^ Studies with NOS score ≥ 6 were considered to be high quality studies.

### 2.4 Statistical analysis

All pooled analyses were performed using STATA 12.0 software (Stata, College Station, TX, USA). HRs and the corresponding 95% CIs were used to evaluate the association between plasma fibrinogen and the prognosis of GC patients; HR > 1 represented a poor prognosis in patients with high plasma fibrinogen. Odds ratios (ORs) with 95% CIs were utilized to assess the relationship between plasma fibrinogen and clinicopathological features; OR > 1 indicated that elevated plasma fibrinogen was correlated with advanced tumor stage, lymph node metastasis, distant metastasis, deeper tumor invasion, poor differentiation, and high carcinoembryonic antigen. Statistical heterogeneity across studies was analyzed using the *Q* statistic test and *I*^*2*^ test. Significant heterogeneity was defined when *P* ≤ .05 and *I*^*2*^ ≥ 50%, and the random effects model was applied. Otherwise, a fixed-effects model was used (*P* > .05 and *I*^*2*^ < 50%). Visual funnel plots and Egger test were used to explore potential publication bias. If significant bias was found, we conducted trim and fill analysis. In addition, sensitivity analysis was also performed to validate the stability of the combined results. A two-tailed *P* value less than .05 was considered statistically significant.

## 3 Results

### 3.1 Study characteristics

**Figure 1:**
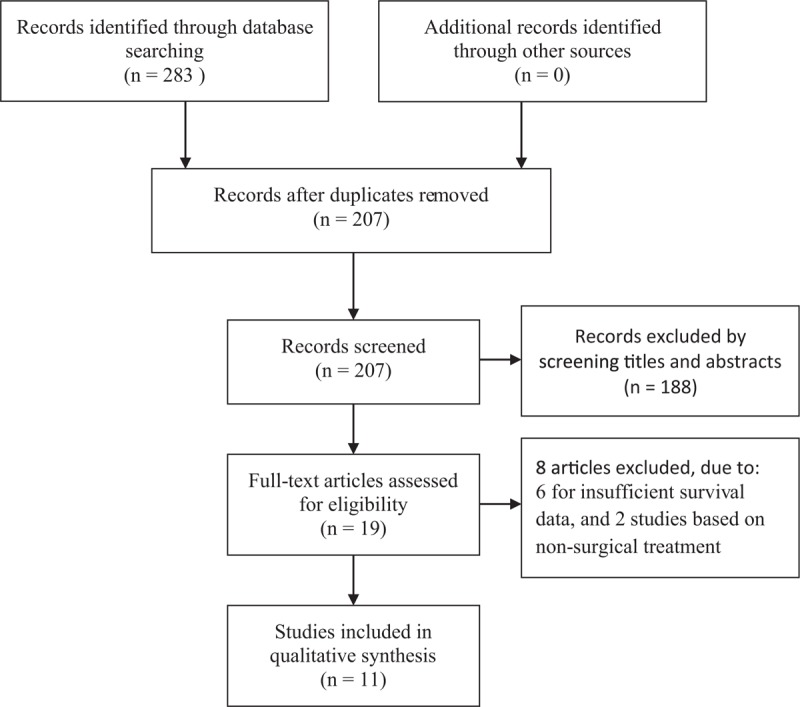
Flow chart of study selection.

A total of 283 records were initially retrieved after searching the databases according to the abovementioned criteria. After removing the duplicate records, 188 studies were directly excluded by scanning the titles and abstracts. In the remaining 19 studies, 8 of these studies were eliminated for the following reasons: 6 for insufficient survival data and 2 studies based on non-surgical treatment. Finally, 11 articles from 3 countries (China, Japan, and Korea) were eligible for this meta-analysis, which were published between 2012 and 2018 (Fig. 1).^[11–15,19–24]^ The main characteristics of the enrolled studies are shown in Table 1. All included studies involving 8315 patients, were retrospective and explored the prognostic role of plasma fibrinogen on OS, 2 of which reported the prognostic impact of plasma fibrinogen on recurrence-free survival (RFS). The cut-off values for fibrinogen ranged from 3.3 to 407 mg/dl. Of these studies, 4 reported on a mixture of non-metastatic and metastatic patients, while 7 reported on non-metastatic patients only. HRs and 95% CIs for OS were directly provided in 8 studies and extracted from Kaplan–Meier survival curves in the other studies. According to the NOS, all included articles were of high quality (score ≥ 6), with a mean of 6.6 (Table 2).

**Table 1:**
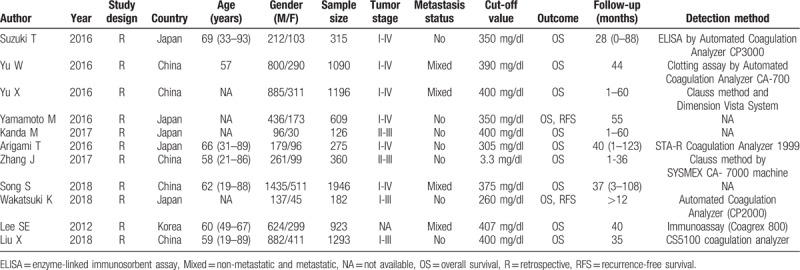
Main characteristics of all included studies.

**Table 2:**
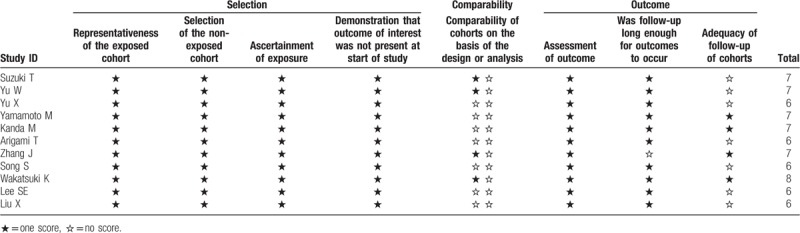
Quality assessment of included studies based on the Newcastle–Ottawa Scale.

### 3.2 Impact of plasma fibrinogen on OS

**Figure 2:**
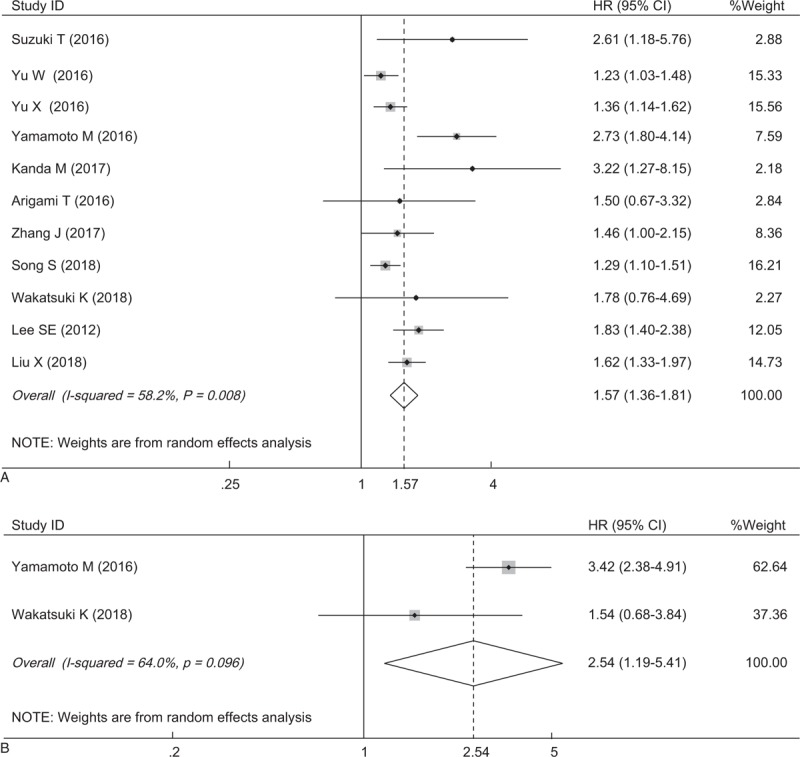
Forest plot showing the pooled HR for the association between elevated plasma fibrinogen and OS (A) or RFS (B) in GC patients. GC = gastric cancer, HR = hazard ratio, OS = overall survival, RFS = recurrence-free survival.

All eligible studies evaluated the association between plasma fibrinogen and the OS in GC. Because of significant heterogeneity (*P* = .008, *I*^*2*^ = 58.2%), a random-effects model was used to pool the HRs. The pooled results indicated that high plasma fibrinogen was significantly associated with shorter OS in GC patients (HR = 1.57, 95% CI: 1.36–1.81, *P* < .001) (Fig. 2A). To detect potential heterogeneity, subgroup analyses were conducted by country, sample size, metastasis status, cut-off value and NOS score. The subgroup analysis according to country demonstrated a positive correlation between elevated plasma fibrinogen and worse OS in Chinese patients (HR = 1.36; 95% CI: 1.25–1.48; *P* < .001), and Japanese patients (HR = 2.43, 95% CI: 1.80–3.27; *P* < .001). In the subgroup analysis of metastasis status, high plasma fibrinogen predicted shorter OS in patients with mixed metastasis (HR = 1.37; 95% CI: 1.20–1.58; *P* < .001) and in patients without metastasis (HR = 1.77; 95% CI: 1.52–2.06; *P* < .001). Similarly, the sample size subgroup analysis indicated that increased plasma fibrinogen was closely associated with poor OS in “ < 1000” group (HR = 1.92; 95% CI: 1.62–2.30; *P* < .001) and in “≥1000” group (HR = 1.35; 95% CI: 1.24–1.48; *P* < .001). Notably, there was no significant heterogeneity in each of these 2 groups. Moreover, statistically significant pooled HR values > 1 were also consistently calculated in subgroup analyses stratified by cut-off value and NOS score (Table 3).

**Table 3:**
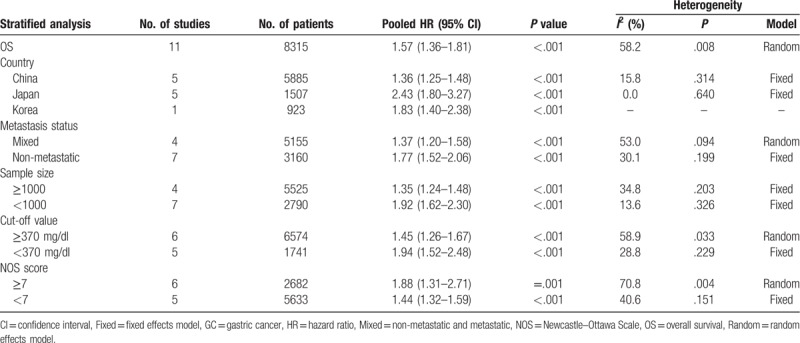
Subgroup analysis of pooled HRs for OS in GC patients with elevated plasma fibrinogen.

### 3.3 Impact of plasma fibrinogen on RFS

Only 2 studies including 791 cases explored the relationship between plasma fibrinogen and RFS. Since significant heterogeneity was observed among the studies (*I*^*2*^ = 64.0%; *P* = .096), the random-effects model was applied. The pooled results showed that RFS was significantly worse in GC patients with high plasma fibrinogen than in patients with low plasma fibrinogen (HR = 2.54; 95% CI: 1.19–5.41, *P* = .016) (Fig. 2B).

### 3.4 Association between plasma fibrinogen and clinicopathological features

**Figure 3:**
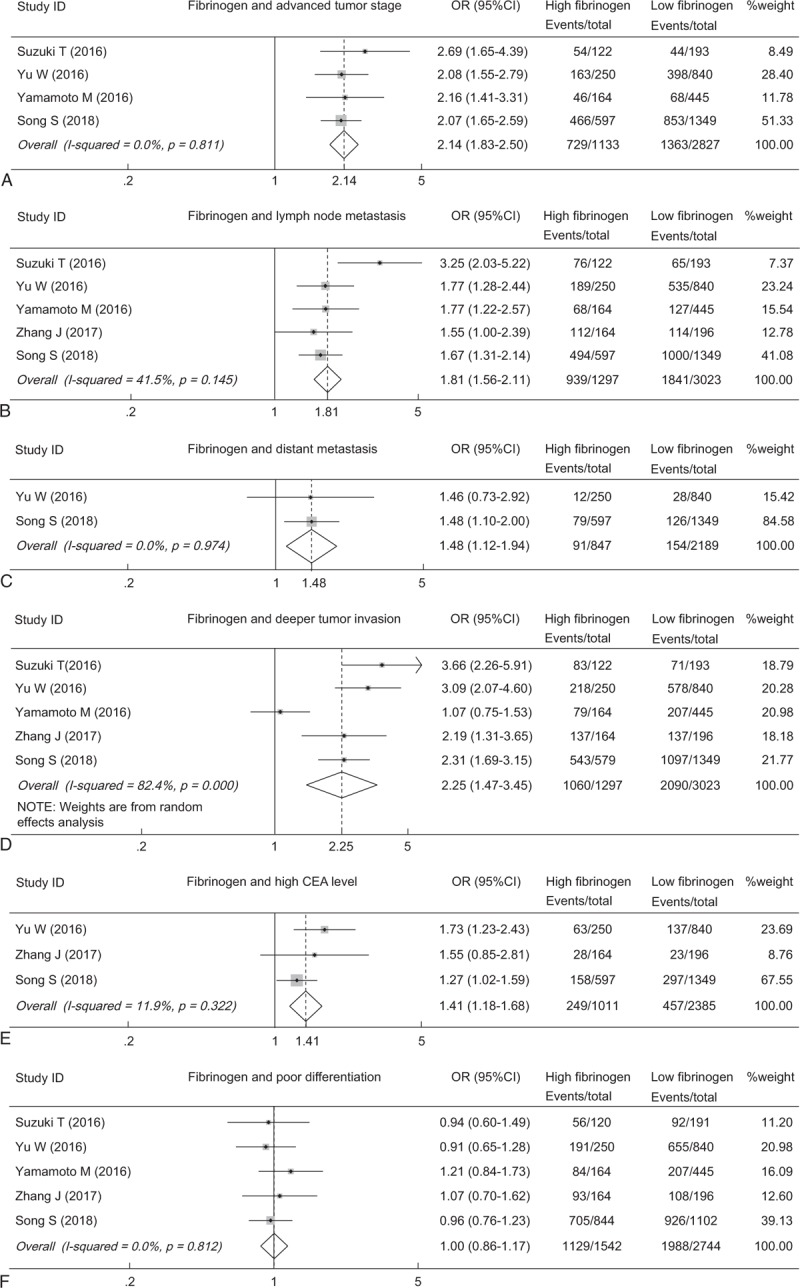
Forest plot showing the pooled OR for the association between elevated plasma fibrinogen and the tumor stage (A), lymph node metastasis (B), distant metastasis (C), tumor invasion (D), CEA (E) and differentiation grade (F). CEA = carcinoembryonic antigen, OR = odds ratio.

To investigate the impact of plasma fibrinogen on clinical characteristics, we identified 6 clinical factors in GC. The pooled results revealed that elevated plasma fibrinogen was significantly correlated with advanced tumor stage (OR = 2.14, 95% CI: 1.83–2.50, *P* < .001) (Fig. 3A), lymph node metastasis (OR = 1.81, 95% CI: 1.56–2.11, *P* < .001) (Fig. 3B), distant metastasis (OR = 1.48, 95% CI: 1.12–1.94, *P* = .005) (Fig. 3C), deeper tumor invasion (OR = 2.25, 95% CI: 1.47–3.45, *P* < .001) (Fig. 3D), and high carcinoembryonic antigen (CEA) (OR = 1.41, 95% CI: 1.18–1.68, *P* < .001) (Fig. 3E). However, no significant association was observed between plasma fibrinogen and the differentiation grade (OR = 1.00, 95% CI: 0.86–1.17, *P* = .967) (Fig. 3F). The detailed results for the relationship between plasma fibrinogen and clinicopathological features were shown in Supplemental Table 1.

### 3.5 Sensitivity analysis

**Figure 4:**
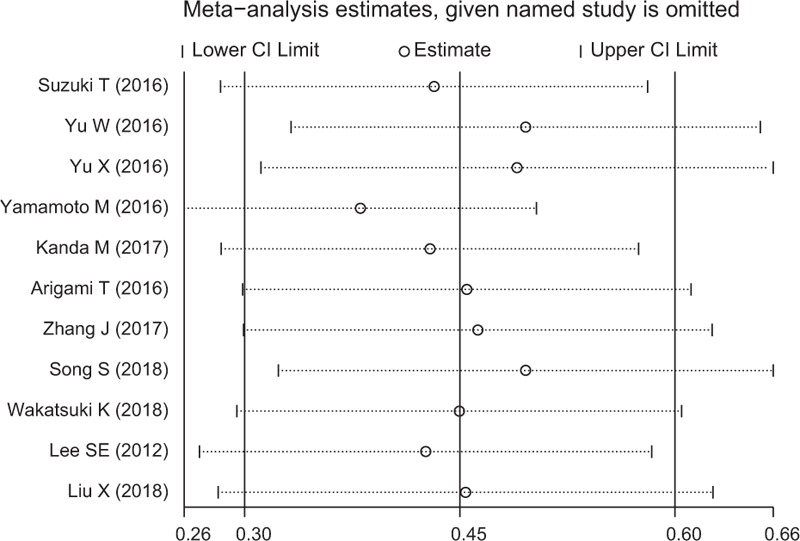
Sensitivity analysis on the association between elevated plasma fibrinogen and OS in GC patients. GC = gastric cancer, OS = overall survival.

Sensitivity analysis were performed to assess the reliability of overall results for OS in GC. The pooled HRs did not significantly vary when excluding any individual study, indicating the robustness of our results (Fig. 4).

### 3.6 Publication bias

**Figure 5:**
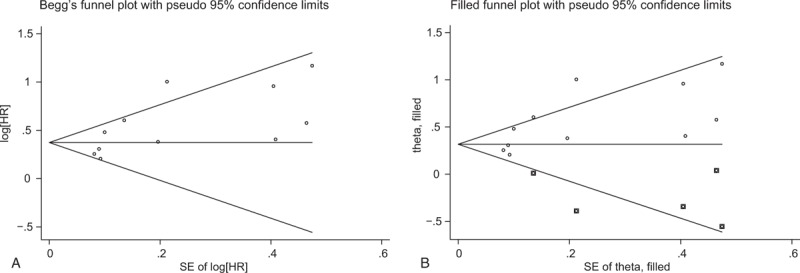
Begg funnel plots evaluating potential publication bias for OS without trim and fill analysis (A) and for OS with trim and fill analysis (B). OS = overall survival, diamonds = included studies, diamonds in squares = presumed missing studies.

Publication bias was evaluated using Begg funnel plot and Egger regression test. The obvious asymmetry of Begg funnel plot provided evidence of publication bias for OS (Fig. 5A), which was confirmed by Egger test (*P* = .03). We then conducted trim and fill analysis (Fig. 5B). The combined HRs were recalculated and still indicated a significant relationship between high plasma fibrinogen and poor OS in GC patients (HR = 1.37; 95% CI: 1.17–1.61, *P* < .001). Therefore, the publication bias in this meta-analysis did not obviously affect the stability of the result.

## 4 Discussion

Recent studies have shown that hyperfibrinogenemia is consistently linked with a large number of malignancies, including gastric cancer.^[25,26]^ Plasma fibrinogen is increasingly recognized as a useful risk factor affecting clinical outcome and tumor progression. However, until now, few meta-analyses have been conducted to evaluate the prognostic significance of plasma fibrinogen in GC patients.

The present meta-analysis included a total of 11 eligible studies enrolling 8315 patients. The pooled results verified that elevated plasma fibrinogen significantly predicted poor OS and RFS in GC. In this meta-analysis, 2 studies by Wakatsuki and Zhang reported the conflicting results that plasma fibrinogen was not an independent prognostic factor for GC patients.^[14,15]^ The possible explanation for this inconsistency was that the cut-off value for hyperfibrinogenemia in these 2 studies was defined as a lower fibrinogen concentration compared with other 9 studies, which may overestimate the number of GC patients with relatively high plasma fibrinogen. Thus, the precision of plasma fibrinogen as a prognostic biomarker in GC may be affected. In addition, we conducted subgroup analyses according to clinical characteristics, due to marked heterogeneity among these cohorts. The subgroup analyses revealed that the significant association between high plasma fibrinogen and worse OS of GC patients was unaffected by country, sample size, cut-off value and NOS score, although the heterogeneity could not be fully eliminated. When stratified by sample size for OS, no significant heterogeneity was observed either in the “<1000” or “≥1000” group. Thus, the sample sizes in different studies might be the main source of heterogeneity. Notably, the metastasis status subgroup analysis also demonstrated that the impact of high plasma fibrinogen on adverse survival remained substantial in various metastatic settings, including non-metastatic and mixed metastatic GC. Moreover, we further explored the correlation between plasma fibrinogen and clinicopathological factors. The results implied that a high fibrinogen level was closely correlated with advanced tumor stage, lymph node metastasis, distant metastasis, deeper tumor invasion and high CEA level. Based on these findings, we speculated that plasma fibrinogen might largely affect the tumor progression, especially advanced tumor stage and metastasis, subsequently leading to short-term recurrence and poor survival in patients with GC.

To date, the potential mechanisms underlying the relationship between high plasma fibrinogen and poor prognosis in GC are still elusive. It has been assumed that cross-talk may exist between the inflammatory response and tumor progression.^[27–29]^ Previous studies demonstrated that fibrinogen might regulate the inflammatory process by inducing monocytes to produce TNF-α and IL-6, which are vital multifunctional cytokines associated with tumor growth and patient survival.^[30–32]^ Fibrinogen also interacts with other cell types such as leukocytes and platelets, and thus exerts its pro-inflammatory and protumor effects in multiple ways.^[33–35]^ On the other hand, fibrinogen might actively participate in cancer progression by promoting tumor cell proliferation, metastasis and angiogenesis. Zhang et al verified that fibrinogen-like-protein 1 (FGL1), a member of the fibrinogen family, was significantly increased in GC cell lines and functioned as a potential mediator of cell proliferation and metastasis.^[36]^ Elevated fibrinogen levels can stimulate tumor cell invasion and migration by inducing the epithelial-mesenchymal transition (EMT), which is considered a key step of aggressive metastasis that is characterized by aberrant expression of vimentin and E-cadherin.^[37]^ Multiple studies by Sahni et al indicated that fibrinogen can facilitate angiogenesis and tumor cell growth by binding to fibroblast growth factor-2 (FGF-2) and vascular growth factor (VEGF), and provides protection for these factors against proteolysis.^[38–40]^ In mouse models, the deletion of fibrinogen reduces aggressive tumor growth and metastasis potential.^[41]^ However, an inconsistent result by Palumbo et al revealed that fibrinogen played a vital role in metastatic progression but not in the growth of primary tumor, since no dramatic differences between wild-type and fibrinogen-deficient mice were observed with regard to the growth of subcutaneously transplanted tumors.^[42]^ The author also found that fibrinogen might be not essential for tumor angiogenesis.^[42]^ Thus, more studies are still needed to elucidate the exact mechanisms involved in the adverse prognostic values of plasma fibrinogen in GC.

To the best of our knowledge, this study is the first meta-analysis to comprehensively assess the clinical and prognostic value of plasma fibrinogen in GC. A high plasma fibrinogen level could indicate poor survival and was a potential risk factor related to multiple aggressive pathological factors. Thus, it might serve as a stratified parameter to identify more high-risk patients with GC before treatment. In clinical practice, fibrinogen, as an inexpensive and non-invasive marker, might be suitable for evaluation of tumor metastasis, local recurrence, and individualized treatment. Given its important prognostic role in cancers, plasma fibrinogen may be combined with other conventional tumor biomarkers such as CEA and carbohydrate antigen 19–9 (CA19–9), which could improve its diagnostic specificity and prognostic assessment in GC.

It is worth noting that there are still some deficiencies in our meta-analysis. First, publication bias exists among the included studies, although the recalculated results indicated the reliability of the combined data according to the trim and fill analysis. Second, several HRs and 95% CIs for OS were extracted from the survival curves based on the statistical results of the univariate analysis, which may overestimate effect sizes because of potential confounding factors. Third, all eligible studies were conducted in Asian countries and published in English only, resulting in selection bias. This may limit the application of our findings to other ethnic groups. Fourth, nonuniform cut-off values were used to define a high plasma fibrinogen level, and the detection methods also vary in some studies, both of which may partly contributed to the heterogeneity. Finally, our meta-analysis was based on retrospective studies, only 2 of which explored the correlation between elevated plasma fibrinogen and RFS, so prospective studies that include all ethnic groups with large sample sizes are imperative to further confirm our findings.

In conclusion, this meta-analysis demonstrated that elevated plasma fibrinogen predicted a worse OS and RFS in patients with GC, and it was also clearly correlated with aggressive clinicopathological features. Thus, preoperative plasma fibrinogen might be a promising prognostic biomarker and a therapeutic target in GC.

## Author contributions

**Conceptualization:** Fei Cheng, Youxiang Chen.

**Data curation:** Fei Cheng, Chunyan Zeng, Ling Zeng.

**Formal analysis:** Fei Cheng, Youxiang Chen.

**Funding acquisition:** Chunyan Zeng, Youxiang Chen.

**Methodology:** Fei Cheng, Chunyan Zeng, Ling Zeng, Youxiang Chen.

**Project administration:** Youxiang Chen.

**Software:** Fei Cheng, Chunyan Zeng, Ling Zeng.

**Supervision:** Youxiang Chen.

**Writing – original draft:** Fei Cheng, Chunyan Zeng.

**Writing – review & editing:** Youxiang Chen.

## Supplementary Material

Supplemental Digital Content
